# Predictors of Unfavorable Outcomes in Diabetic Foot Ulcers

**DOI:** 10.3390/diagnostics15233070

**Published:** 2025-12-02

**Authors:** Renata Pauliukienė, Kristina Šutienė, Aistė Čemerkaitė, Jonas Čeponis

**Affiliations:** 1 Department of Endocrinology, Faculty of Medicine, Medical Academy, Lithuanian University of Health Sciences, Eiveniu 2, LT-50161 Kaunas, Lithuania; renata.pauliukiene@lsmu.lt; 2 Department of Mathematical Modeling, Kaunas University of Technology, Studentu 50, LT-51368 Kaunas, Lithuania; kristina.sutiene@ktu.lt; 3 Institute of Endocrinology, Lithuanian University of Health Sciences, Eiveniu 2, LT-50161 Kaunas, Lithuania; aiste.cemerkaite@lsmu.lt

**Keywords:** diabetes mellitus, diabetic foot ulcer, outcomes, risk factors, machine learning analysis

## Abstract

**Background/Objectives**: The aim of this study was to identify systemic, metabolic, and host-related prognostic factors for long-term outcomes in patients with a diabetic foot ulcer (DFU). **Methods**: One hundred patients were selected from a high-risk cohort of 426 individuals with a DFU (January 2021–January 2023) based on predefined inclusion and exclusion criteria. Clinical, laboratory, and imaging data were collected. Outcomes were categorized as favorable (healing) or unfavorable (non-healing, re-ulceration, amputation, or death). Prognostic factors were analyzed using random forest and categorical boosting models, with SHAP values to determine the importance of individual predictors. **Results**: The median age of participants was 65 years (interquartile range [IQR], 57–69.25), and the median duration of diabetes was 18 years (IQR, 12–26). Over a mean 2.1-year follow-up, unfavorable outcomes occurred in 53% of the whole cohort and in 36% of survivors. The strongest predictors of poor prognosis were prior amputation, elevated inflammatory markers, reduced eGFR, and dyslipidemia. Triglycerides showed a U-shaped association with outcomes. A lower BMI and shorter diabetes duration paradoxically were also linked to poorer prognosis. Glycemic control, comorbidities, and local foot characteristics had limited predictive value. **Conclusions**: Long-term DFU prognosis is driven mainly by systemic and host-related factors rather than by ulcer characteristics alone. Inflammation, renal dysfunction, dyslipidemia—particularly triglycerides—and prior amputation were the strongest predictors of unfavorable outcomes.

## 1. Introduction

Diabetic foot disease represents one of the most severe and debilitating complications of diabetes, contributing substantially to morbidity, premature mortality, and escalating healthcare costs worldwide. With the prevalence of diabetes continuing to rise, affecting an estimated 65.6 million individuals in Europe in 2024, with a projected 10% increase by 2050 [1], the global burden of diabetic foot disease is anticipated to grow proportionally.

Over the course of their lifetime, between 19% and 34% of individuals with diabetes will develop a diabetic foot ulcer (DFU) [2,3,4,5]. More than half of these ulcers progress to infection [6], and approximately 20% of moderate-to-severe infections ultimately culminate in amputation [7,8]. Even after apparently successful healing, recurrence is alarmingly frequent: around 40% of patients experience a new ulcer within one year, nearly 60% experience a new ulcer within three years, and up to 65% experience a new ulcer within five years [2].

Mortality in patients with a DFU is markedly elevated, with approximately 50% dying within five years of diagnosis [2,4,5]. The risk of death is more than doubled compared to individuals with diabetes who do not develop foot ulcers [2,9]. The leading causes of death are cardiovascular disease and infection, accounting for almost 50% and 25% of deaths, respectively [4].

Identifying predictors of unfavorable outcomes is essential to enhance risk stratification, guide timely and targeted interventions, and ultimately mitigate the burden of diabetes-related foot complications. Numerous factors have been related to poor outcomes in DFU, including advanced age, male sex, peripheral arterial disease, chronic kidney disease, heart failure, prior cardiovascular events, ulcer severity (including depth, location—especially hindfoot—and presence of infection or ischemia), delayed presentation, and history of previous ulcer or amputation [5,10,11,12,13,14]; however, despite substantial progress in this field, a universally accepted and validated tool capable of reliably predicting outcomes in high-risk DFU patients is still lacking [15,16,17,18,19].

The aim of this investigation was to evaluate clinical, laboratory, and imaging factors associated with unfavorable outcomes in high-risk patients with a DFU. To address this objective, we analyzed a well-characterized cohort of individuals with long-standing diabetes and active foot ulceration, integrating demographic, clinical, laboratory, and imaging data.

## 2. Materials and Methods

### 2.1. Patient Population

For the purpose of this study, a series of 100 patients treated for a DFU that were seen by the multidisciplinary consultation unit at the Department of Endocrinology, Hospital of the Lithuanian University of Health Sciences Kaunas Clinics between January 2021 and January 2023 were evaluated.

During this period, a total of 426 patients were seen at the unit, of whom 115 were assessed dead by June 2024.

The inclusion criteria for the analysis were as follows:

- Disease codes, based on the International Statistical Classification of Diseases and Related Health Problems, Tenth Revision, Australian Modification (ICD-10-AM): E11.73, E10.73, E11.52, E10.52, L97, Z89.4, Z89.5, and Z89.6;
- Confirmed diagnosis of type 1 or type 2 diabetes mellitus;
- Seen by the multispecialty consultation unit at the Department of Endocrinology for a confirmed DFU;
- Age between 18 and 80 years.

Patients were excluded if they had other types of diabetes; were undergoing dialysis for end-stage renal disease (ESRD); had advanced malignancy or immunosuppressive disease; or were receiving immunosuppressant therapy.

For the analysis, 73 patients were reassessed in the clinic in December 2023–June 2024, duration of observation 1.2–3 years (mean 2.1 years), as well as the first visit. Database data were extracted of 27 randomly selected deceased subjects meeting the aforementioned eligibility criteria. All participants provided written informed consent prior to their enrolment in this study.

### 2.2. Data Collection

The following data were collected for each patient: demographic variables (age, gender), diabetes-related variables (type and duration of diabetes, use of antidiabetic medications, previous foot ulcer and/or history of lower limb amputation), and comorbidities (history of stroke, myocardial infarction, presence of arterial hypertension, chronic kidney disease). Collection and documentation of demographic data were performed by an endocrinologist.

Objective clinical parameters included body mass index (BMI), presence of foot deformities, and assessment of diabetic polyneuropathy and peripheral angiopathy. Body mass index (BMI) was calculated as weight in kilograms divided by height in meters squared (kg/m^2^), based on the World Health Organization (WHO) criteria. Weight was measured to the nearest 0.1 kg, with participants wearing light clothing and no shoes, and height was recorded to the nearest 0.1 cm using a stadiometer. Foot deformities were assessed for the presence of hallux valgus, toe deformities, Charcot deformity, and arch abnormalities. Diabetic polyneuropathy was assessed using a 10 g monofilament and a tuning fork in accordance with the International Working Group on the Diabetic Foot (IWGDF) recommendations [20]. Peripheral arterial disease was evaluated by palpation of the a. dorsalis pedis and a. tibialis posterior. The clinical evaluation was performed by a trained diabetes nurse.

Laboratory tests comprised lipid profile (total cholesterol (TC), low-density lipoprotein cholesterol (LDL-C), high-density lipoprotein cholesterol (HDL-C), triglycerides), white blood cell (WBC) count, C-reactive protein (CRP), erythrocyte sedimentation rate (ESR), and estimated glomerular filtration rate (eGFR); glycemic control was assessed by glycosylated hemoglobin (HbA1c). Biochemical and hematological analyses were carried out in the local clinical laboratory using automated analyzers and established assay methodologies, performed according to standardized protocols.

Imaging evaluation consisted of bilateral foot radiographs obtained in two standard projections primarily aimed at detecting radiological signs of osteomyelitis. The X-ray results were coded as 0—not performed, 1—no osteomyelitis, and 2—confirmed osteomyelitis.

The presence and absence of a feature for each categorical variable was coded as 1 and 0, respectively.

### 2.3. Outcome Measures

For this analysis, the outcomes of clinical course of the DFU were dichotomized as favorable or unfavorable. An unfavorable outcome (“event”) was defined as failure to heal of a previously existing ulcer, development of a new ulcer, any level of lower-limb amputation (ranging from major amputations above the ankle to minor foot amputations, including toes), or death.

In the survivor group analysis, the event was an unhealed existing ulcer, development of a new ulcer, or amputation.

Patients were stratified according to clinical outcome, classified as favorable (outcome = 0) or unfavorable (outcome = 1).

### 2.4. Prediction Problem

Consider a dataset with n observations and m features, denoted as $D\;=\;\{\left(x_{i},y_{i}\right)\}$, where $x_{i}\in R^{m}$ corresponds to the input vector of i-th observation, while $y_{i}\in (1,\;\ldots ,\;K)$ denotes its categorical class label for K possible classes, with $i\;=\;\bar{1,n}$. For any given instance $x_{i}$, the machine learning model $h(x_{i})$ produces a prediction ${\hat{y}}_{i}$, typically expressed as either a class probability distribution over K categories or a discrete class label. In this study, two ensemble-based machine learning models were selected to address the prediction of DFU outcome.

The dataset under investigation comprises both categorical and numerical features. To completely employ its structure, categorical boosting (CatBoost) was chosen, as it achieves state-of-the-art predictive accuracy and is specifically designed to process categorical variables through ordered target statistics. Its boosting framework enables capturing complex non-linear relationships, which are often present in medical data [21,22,23]. As an alternative, random forest (RF) was chosen to serve as a robust and interpretable baseline. RF builds multiple decision trees on bootstrapped samples and aggregates their outputs, thereby reducing variance and enhancing stability in noisy or heterogeneous datasets [24,25,26].

#### 2.4.1. Categorical Boosting

CatBoost, introduced by [27], constructs an ensemble of decision trees sequentially, with each tree focusing on correcting the errors of the previous ones. Its objective function is explicitly regularized and can be expressed as follows:

$$L\;=\sum_{i=1}^{n}l(y_{i},{\hat{y}}_{i})+\Omega \left(f\right),$$

where $l\left(y_{i},{\hat{y}}_{i}\right)=-\sum_{k=1}^{K}1\left\{y_{i}=k\right\}log(p_{i,k})$ represents the loss function, with $1\left\{y_{i}=k\right\}$ being an indicator function, and $p_{i,k}=\frac{exp({\hat{y}}_{i,k})}{\sum_{j=1}^{K}exp({\hat{y}}_{i,j})}$ representing a softmax probability of class k. The L2 regularisation penalty is defined as $\Omega \left(f\right)\;=\;\lambda \sum_{j=1}^{T}\omega_{j}^{2},$ where $\omega_{j}$ are the leaf weights and λ is the regularization coefficient. This formulation ensures that CatBoost not only optimizes predictive accuracy but also prevents overfitting, making it well suited for heterogeneous datasets with categorical and numerical features. The balance between accuracy and generalization is achieved through the careful adjustment of several hyperparameters, which directly influence how the model learns from data and controls complexity [28,29]:

Parameters like iterations, depth, and learning_rate affect how quickly the loss is minimized. More specifically, the iterations parameter specifies the number of boosting rounds, where additional trees can improve predictive accuracy but also increase the risk of overfitting. The learning_rate η controls the optimization step size, with lower values producing more stable and reliable probability estimates, though requiring more iterations to converge. The depth parameter determines the maximum depth of individual trees: deeper trees are able to capture complex patterns in patient data but may overfit, making this parameter essential for balancing accuracy with generalization;

l2_leaf_reg and random_strength directly regularize the objective, smoothing solutions and avoiding overfitting. In healthcare data, where sample sizes are often modest, this regularization improves robustness against spurious associations. By using random_strength, higher randomness when selecting tree splits is enabled, thereby improving its ability to generalize across diverse patient populations;

cat_features influences how categorical data enter the optimization process. Unlike many other machine learning algorithms that require manual preprocessing (e.g., one-hot encoding), CatBoost can process categorical data using its ordered boosting scheme, which allows the capture of meaningful patterns in categorical variables without exploding dimensionality as in one-hot encoding;

early_stopping_rounds and num_folds ensure that progress in the objective function indicates meaningful learning rather than spurious patterns from noise. In particular, the parameter early_stopping_rounds specifically monitors the validation loss $L_{val}$, while the parameter num_folds indicates the number of folds used in cross-validation by helping to evaluate the model’s robustness across multiple subsets and reduce the risk of biased performance estimates.

#### 2.4.2. Random Forest

Random forest (RF), introduced by Breiman [30], is an ensemble learning method based on the principle of bootstrap aggregation (bagging) applied to decision trees. It is widely used in medical diagnostics due to its robustness, interpretability, and ability to handle heterogenous datasets [31,32,33]. Each tree is trained independently on a random sample, with further randomness introduced by selecting a random subset of features at each split. Instead of minimizing a global objective with explicit regularization, RF relies on split criteria such as Gini impurity or information gain at each node, such as

$$Gini=1\;-\;\sum_{k=1}^{K}p_{k}^{2},$$

where *p_k_* is the proportion of samples of class k at the node. The ensemble averaging reduces variance and prevents overfitting. RF performance and generalization are strongly influenced by a set of hyperparameters that control the ensemble size, tree complexity, and randomness introduced during training. The key parameters in balancing predictive power and overfitting prevention are as follows [34,35]:

- *n_estimators* are used to control the ensemble size. In particular, it defines the number of decision trees in the forest. A larger number of trees generally improve stability and reduce variance but increase the computation time;
- *max_depth*, *min_samples_split*, and *min_samples_leaf* are the parameters used to determine the tree complexity. Using these parameters, the maximum depth of each tree, the minimum number of samples required to split a node, and the minimum number of samples allowed in a terminal leaf are specified, respectively. Collectively, these settings determine how each decision tree in the forest is constructed. By constraining depth, splits, and leaf size, the parameters are used to prevent the model from overfitting while maintaining sufficient flexibility to identify certain patterns in the data;
- *max_features* and *bootstraps* are used to control diversity and randomness. By regulating the number of features used at each split, the diversity and correlation of trees are controlled. In comparison, the randomness is introduced using bootstrap through resampling, which allows the reduction of variance and stabilizes the overall model performance.

#### 2.4.3. Fine-Tuning of Model Parameters

To optimize the listed parameters of both CatBoost and RF, tree-structured Parzen estimator (TPE) [36,37] was implemented. TPE is a form of Bayesian optimization that models the hyperparameter search space probabilistically. First, several trials are split into good and poor configurations according to the objective metric (e.g., balanced accuracy in this study). Then, two probability density functions are constructed using Gaussian mixture models: *l*(*x*)—likelihood of hyperparameters among the good configurations, *g*(*x*)—likelihood of hyperparameters among all other configurations. A configuration of new parameters is selected by maximizing the likelihood ratio:

$$x^{*}\;=\;arg\underset{x}{max}\frac{l(x)}{g(x)}.$$

This ensures that values similar to those in the good configurations are sampled more frequently, focusing the search on promising regions of the parameter space. With each trial, the density functions of *l*(*x*) and *g*(*x*) are updated. Thus, as trials continue, TPE gradually adjusts its sampling strategy to favor regions of the hyperparameter combinations observed for better performance, thereby converging efficiently toward optimal configurations.

#### 2.4.4. Performance Metrics

To assess the generalization performance of the trained model and its predictive ability on unseen data, k-fold cross-validation (CV) was applied [38]. This approach evaluates model stability by partitioning the dataset into k equal folds by training on k−1 folds and validating on the remaining folds. By using multiple splits, k-fold CV reduces the risk of biased evaluation results caused by a single train–test division, thereby providing a more reliable estimate of predictive performance.

The evaluation of binary classification models is measured using metrics derived from the confusion matrix [39], which compares predicted outcomes against actual outcomes. Suppose true positives (TP) represent correctly predicted positive cases, true negatives (TN) denote correctly predicted negative cases, false positives (FP) show the number of negative cases that were incorrectly classified as positive, and false negatives (FN) show positive cases mistakenly classified as negative. The detailed formulas for all metrics are provided in Supplementary Material S1.

In diagnostic tasks, recall ensures actual outcomes are not overlooked, precision ensures positive predictions are trustworthy, and balanced accuracy together with AUC provide a robust performance metric in the presence of class imbalance. Using multiple complementary metrics allows for a more comprehensive assessment of model performance.

#### 2.4.5. Explanations Using SHAP Values

To improve the interpretability of the CatBoost and RF models, we applied SHapley Additive exPlanations (SHAP). This is a state-of-the-art approach that provides both local interpretability (at the level of individual predictions) and global interpretability (feature importance). SHAP decomposes each prediction into additive contributions of individual features based on cooperative game theory [40,41]. This approach is particularly valuable for identifying the key factors driving model decisions, thereby supporting model validation and deepening the understanding of the underlying clinical phenomena.

In particular, for a prediction *h*(*x*), the SHAP value is decomposed as

$$h\left(x\right)=\phi_{0}+\sum_{j=1}^{m}\phi_{j},$$

where $\phi_{0}$ denotes the model’s average prediction across the dataset, while $\phi_{i}$ represents the SHAP value associated with feature i, with m being the total number of input features. Each reflects the marginal contribution of feature i to the prediction averaged across all possible feature subsets [42]. The exact contribution of a given feature j is given by

$$\begin{array}{c} \phi_{j}=\sum_{S\subseteq Nj\}\frac{|S|!(|N\left|-\right|S|-1)!}{|N|!}\;\left(h_{S\cup \{j\}}-{\;h}_{S}\right)}, \end{array}$$

where S⊆Nj} represents a subset of all features excluding feature j, N is the full set of features, $h_{S\cup \{j\}}$ denotes the model prediction when feature j is included in subset S, and ${\;h}_{S}$ corresponds to the prediction based solely on features in S. As the computation of exact $\phi_{j}$ values is very computationally expensive, tree-based models, such as CatBoost and random forest, enable efficient computation of SHAP values by exploiting the internal structure of decision trees [43].

### 2.5. Ethical Considerations

Approval by the Kaunas Regional Biomedical Research Ethics Committee for the biomedical study (No. BE-2-22) was received 22 March 2023.

## 3. Results

**Figure 1:**
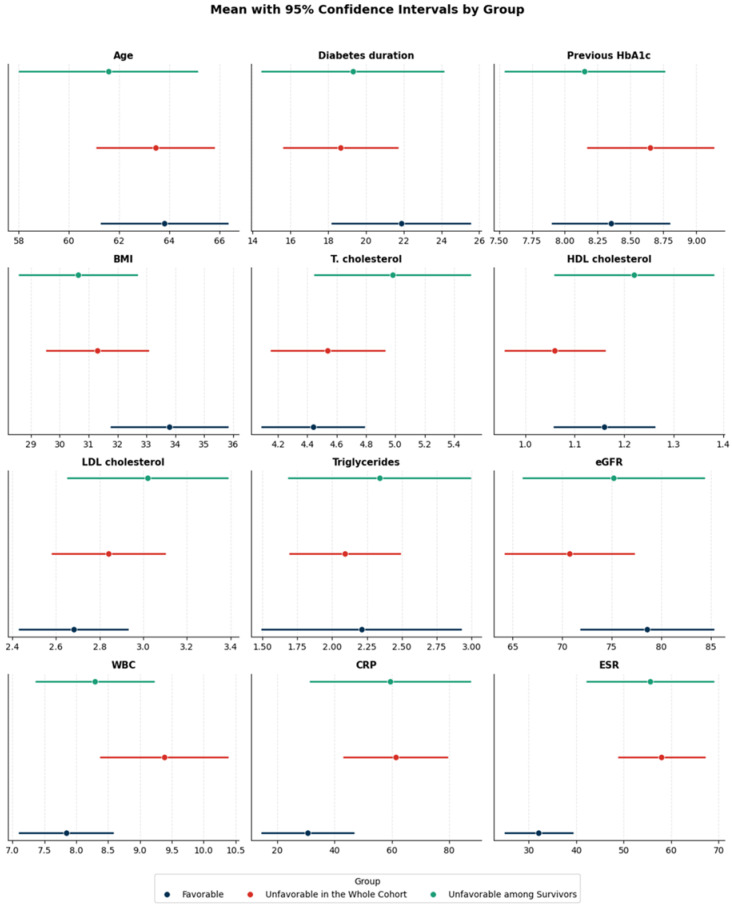
The means with 95% confidence intervals (CIs) across the subject groups: favorable, unfavorable in the whole cohort, and unfavorable among survivors. Each subplot corresponds to a single variable. Abbreviations: HbA1c—glycosylated hemoglobin; BMI—body mass index; T. cholesterol—total cholesterol; HDL—high-density lipoprotein; LDL—low-density lipoprotein; eGFR—estimated glomerular filtration rate; WBC—white blood cell count; CRP—C-reactive protein; ESR—erythrocyte sedimentation rate.

**Figure 2:**
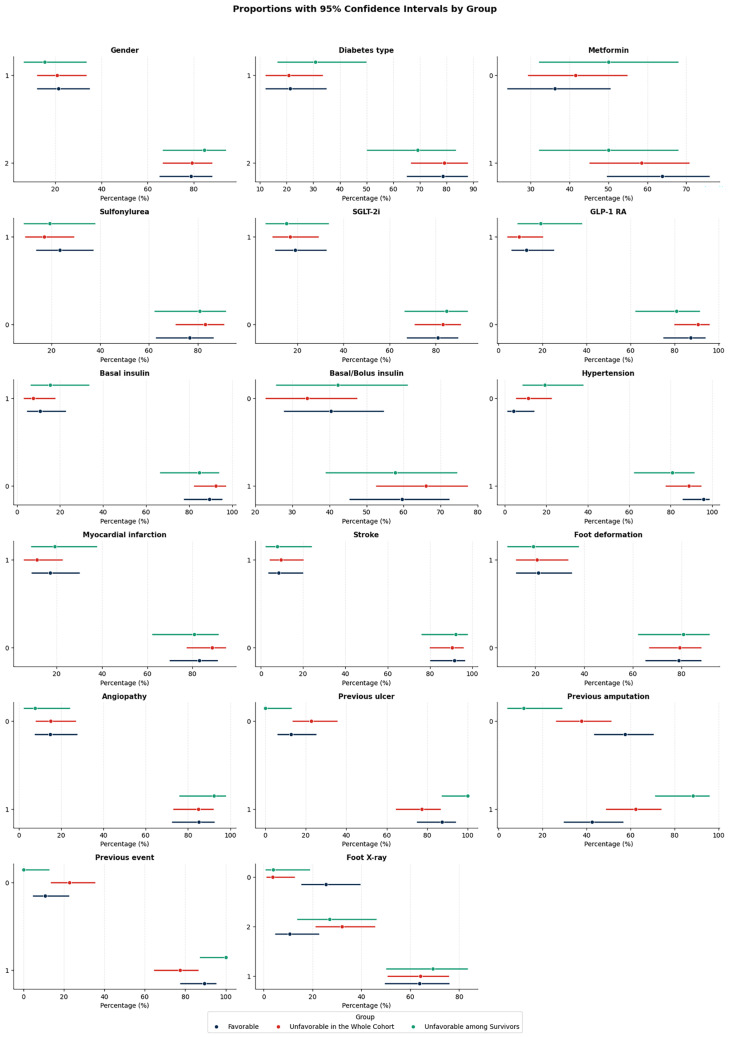
Comparison of proportions with 95% confidence intervals (CIs) for categorical variables across the groups: favorable, unfavorable in the whole cohort, and unfavorable among survivors. A value of 1 indicates the presence of the trait. Abbreviations: SGLT-2i—sodium–glucose cotransporter 2 inhibitors; GLP-1RA—glucagon-like peptide-1 receptor agonists; Foot X-rays—plain foot radiographs obtained in two standard projections (anteroposterior and lateral) to assess the presence of osteomyelitis.

Over the observation period (mean duration 2.1 years), data from 100 patients were available for analysis: a total of 27 died before the outcome visit, and 73 were reassessed in the clinic. The demographics of the patients are presented in Table 1, while the comparisons for continuous and categorical variables in both cohorts by outcome are shown in Figure 1 and Figure 2, respectively.

The median age was 65 years (interquartile range [IQR], 57–69.25; range, 41–77 years), and the duration of diabetes was 18 (IQR, 12–26) years, reflecting a population with long-standing and advanced disease. The majority of patients were overweight or obese, and the glycemic control was generally suboptimal, with a mean HbA1c level of 8.3 (IQR 7.25–9.6)%. Lipid parameters demonstrated notable variability: LDL cholesterol (2.60 [IQR, 2.03–3.37] mmol/L) and total cholesterol (4.35 [IQR, 3.45–5.35] mmol/L) were moderately elevated in relation to the patients’ high cardiovascular risk, underscoring the prevalent burden of dyslipidemia in this cohort. HDL cholesterol levels varied widely, while triglyceride concentrations demonstrated pronounced dispersion (range, 0.4–18.0 mmol/L), indicating substantial metabolic heterogeneity within the research population. The median eGFR of 74.9 (IQR, 59.4–93.15) mL/min/1.73 m^2^ indicates that the majority of patients exhibited mildly reduced yet predominantly preserved renal function. Inflammatory markers (CRP, ESR, WBC) were substantially elevated and highly variable, suggesting pronounced systemic inflammation and possible underlying infection. Overall, the study cohort was characterized by long-standing diabetes, poor metabolic control, obesity, variable degrees of renal dysfunction, and high systemic inflammatory burden—features consistent with a population at increased risk of adverse DFU outcomes.

Unfavorable outcomes were observed in 53 subjects (53%) of the overall cohort and in 26 (36%) of the survivors. Of the latter, ulcers failed to heal or recured in seven subjects (26.9%), while fourteen (53.8%) and five (19.2%) patients underwent minor and major amputations, respectively. When stratified by diabetes type, unfavorable outcomes were observed in 38.1% of patients with type 1 diabetes (T1D) and 53.2% of those with type 2 diabetes (T2D) in the overall cohort. Among survivors, these outcomes occurred in 44.4% of T1D and 32.7% of T2D patients.

In the outcome comparison between the two cohorts, only three groups are presented, as the favorable outcome groups overlapped.

While the metrics in the favorable outcome group could largely be interpreted as the most positive, for the majority of variables, only modest numerical differences were observed, with a substantial overlap in intervals indicating no statistical significance. Interestingly, age, diabetes duration, and BMI trended to be the highest in this group. Inflammatory markers (WBC, CRP, ESR) demonstrated the greatest separation among the groups, especially for ESR.

For categorical variables, the use of metformin and hypertension tended to differ between the favorable and unfavorable groups, though some overlap of confidence intervals is observed. Comparatively, myocardial infarction appeared more common in the unfavorable groups. Previous amputations, previous events, and previous ulcerations were more common in the unfavorable groups, especially among survivors. To sum up, the most discriminative categorical predictors of unfavorable outcomes are previous ulcer, previous amputation, foot deformation, and previous event (any previous ulcer or amputation). These risk factors strongly differentiate the patient groups, while variables such as hypertension, stroke, myocardial infarction, and angiopathy also contributed but with more overlap.

### Risk Factor Analysis and Modelling

**Figure 3:**
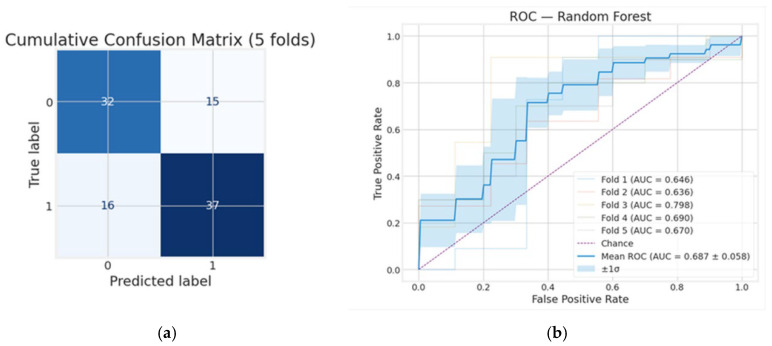
Whole cohort: (**a**) cumulative confusion matrix and (**b**) receiver operating characteristic (ROC) curves for the random forest model. True labels are shown on the *y*-axis, and predicted labels are shown on the *x*-axis.

As shown in Figure 3, the cumulative confusion matrix summarizes the random forest model’s performance across 5-fold cross-validation, showing 32 true negatives, 37 true positives, 15 false positives, and 16 false negatives. These results indicate that sensitivity (69.8%) and specificity (68.1%) are closely balanced, suggesting the model performs comparably in predicting both positive and negative cases. Similarly, precision (71.2%) and an F1-score of 0.705 indicate a reasonable balance between correctly identifying true positives and minimizing false positives. The balanced accuracy (0.689) further confirms a moderate generalization across classes. In the same vein, ROC analysis over k = 5 folds suggests that the RF model has a moderate predictive ability in distinguishing between unfavorable and favorable outcomes. The differing values of AUC values among folds reflect variability in model performance across data splits.

**Figure 4:**
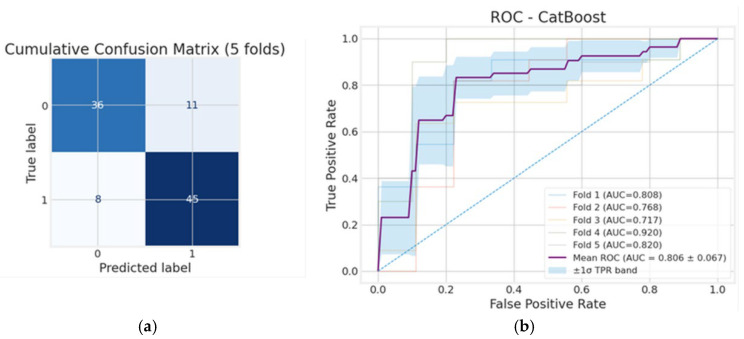
Whole cohort: (**a**) cumulative confusion matrix and (**b**) receiver operating characteristic (ROC) curves for the CatBoost model.

Figure 4 illustrates the CatBoost model’s performance for the whole cohort. In panel (a), the cumulative confusion matrix shows the aggregated results across 5-fold cross-validation, where 36 true negatives and 45 true positives were correctly identified alongside 11 false positives and 8 false negatives. This indicates that CatBoost achieves an overall accuracy of 81.0%, with sensitivity (84.9%) slightly exceeding specificity (76.6%), suggesting stronger performance in detecting unfavorable outcomes. Precision (80.4%), together with an F1-score of 0.826 and a balanced accuracy of 0.808, reflects a strong balance between minimizing false positives and correctly identifying true unfavorable cases. This suggests that the CatBoost model, being optimized for categorical variables, showed higher predictive values and, thus, was used for further risk factor analysis in the whole sample as well as the survivor-only analysis. As shown in Figure 4b, the CatBoost classifier achieved an AUC of 0.806 on average, indicating solid predictive ability in distinguishing unfavorable from favorable outcomes across the full patient set. While there is some variability between folds (ranging from 0.717 to 0.920), the overall performance remains above chance, confirming that CatBoost generalizes well across different subsets of patient data and performs better than RF.

**Figure 5:**
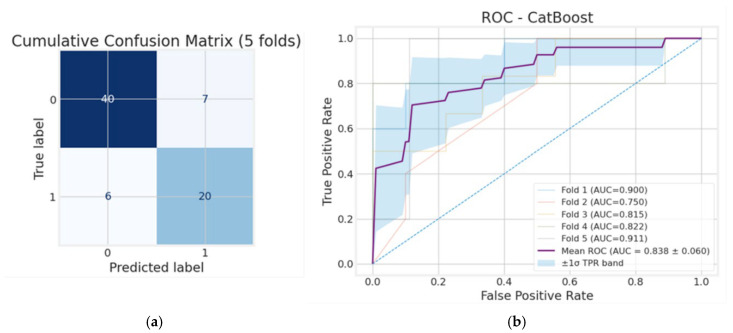
Survivors’ cohort: (**a**) cumulative confusion matrix and (**b**) receiver operating characteristic (ROC) curves for the CatBoost model.

Figure 5 depicts the performance of the CatBoost model in the survivor cohort. In Figure 5a, the cumulative confusion matrix summarizes the 5-fold cross-validation results, showing 40 correctly identified negatives and 20 correctly identified positives alongside seven false positives and six false negatives. It shows that CatBoost achieved an overall accuracy of 82.2%, with relatively balanced performance (0.810) across both classes. Specificity (85.1%) is slightly higher than sensitivity (76.9%), suggesting the model is more effective at correctly identifying negative cases while still maintaining good performance in detecting unfavorable outcomes. The precision (74.1%) and F1-score (0.755) indicate a reasonable trade-off between minimizing false unfavorable cases and maximizing true detection of unfavorable cases. In Figure 5b, the mean AUC of 0.838 demonstrates the CatBoost model’s strong discriminative power between unfavorable and favorable cases. Despite some fold-to-fold variability (ranging from 0.750 to 0.911), the performance remains robustly above chance, suggesting that CatBoost generalizes well to this patient group and captures clinically relevant predictive patterns.

**Figure 6:**
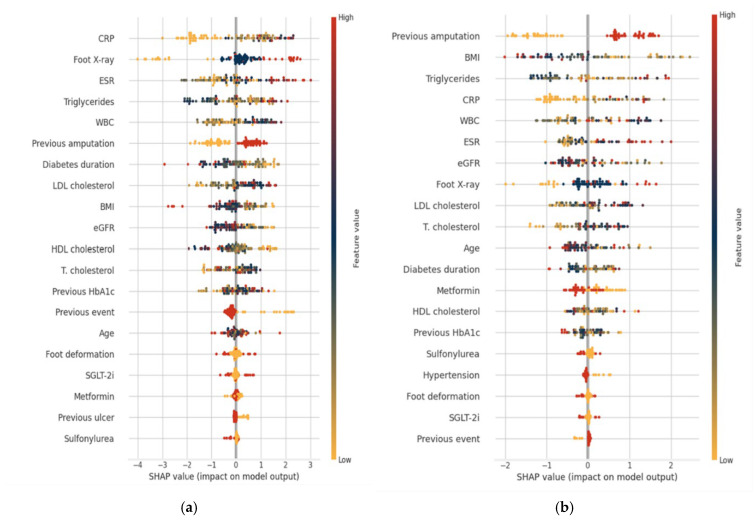
SHAP (SHapley Additive exPlanations) summary plots for the CatBoost model: (**a**) whole cohort and (**b**) survivors’ cohort. Each point represents a single patient. The horizontal axis displays SHAP values, which indicate both the direction and magnitude of each feature’s impact on the prediction of unfavorable outcomes. Features are ranked in descending order of overall importance, with the most influential variables presented at the top. The color of each point represents the original feature value (red = high, blue = low). Abbreviations: HbA1c—glycosylated hemoglobin; BMI—body mass index; T. cholesterol—total cholesterol; HDL—high-density lipoprotein; LDL—low-density lipoprotein; eGFR—estimated glomerular filtration rate; WBC—white blood cell count; CRP—C-reactive protein; ESR—erythrocyte sedimentation rate; SGLT-2i—sodium–glucose cotransporter 2 inhibitors; Foot X-ray—plain foot radiographs obtained in two standard projections (anteroposterior and lateral) to assess the presence of osteomyelitis.

SHAP summary plots, presented in Figure 6, illustrate the contribution of individual features in both the full cohort and survivor analyses, offering a clear visual representation of how varying feature levels impact model predictions. In the full cohort analysis (Figure 6a), risk factors for unfavorable outcomes, including non-healing, re-ulceration, amputation, and death, are summarized. The strongest predictors identified were the elevated inflammatory markers (CRP, WBC, ESR), radiographic evidence of osteomyelitis, a U-shaped association with triglycerides (both low and high levels), prior amputation, shorter diabetes duration, higher LDL-C, lower BMI, and reduced eGFR. In the survivor-only analysis (Figure 6b), prior amputation was the most powerful predictor, followed by lower BMI, U-shaped triglycerides, elevated inflammatory markers (CRP, WBC and ESR), reduced eGFR, radiographic evidence of osteomyelitis, higher total and LDL-C cholesterol, younger age, and shorter diabetes duration. Among the medications evaluated, only metformin demonstrated a consistent, albeit modest, protective association in survivors, with no corresponding effect in the overall cohort, whereas other antihyperglycemic agents had minimal impact. In contrast, glycemic control, comorbidities, and foot examination findings appeared to have less prognostic influence than the aforementioned factors in our analysis.

**Figure 7:**
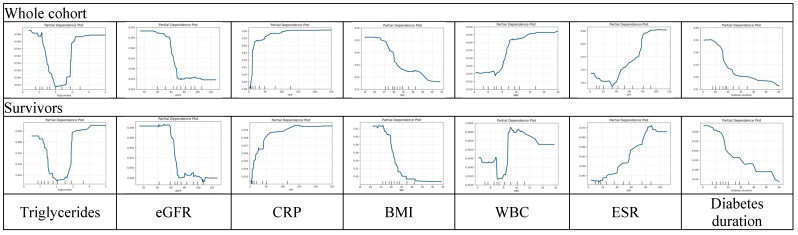
Partial dependence plots (PDPs) illustrating the relationship between the values of key predictors and the probability of unfavorable outcomes. PDPs show the marginal effect of selected individual features on the model’s predicted probability of unfavorable outcomes while averaging out the effects of all other variables. The horizontal axis represents the feature value, and the vertical axis shows the corresponding predicted probability. Abbreviations: BMI—body mass index; eGFR—estimated glomerular filtration rate; WBC—white blood cell count; CRP—C-reactive protein; ESR—erythrocyte sedimentation rate.

The prognostic impact of the risk factors was illustrated using partial dependence plots, which provide a detailed visualization of each factor’s independent effect on the predicted outcome and yield deeper insight into their prognostic relevance (Figure 7). This analysis reinforced a U-shaped pattern for triglycerides, where the risk is higher at both low and high triglyceride values. This pattern is present in both cohorts. Other factors followed the previously described tendencies, with some variability seen in WBC for the Survivor group, where the risk was the lowest in the normal range, with a slight elevation in the low WBC range and significantly elevated risk at higher levels.

## 4. Discussion

Previous studies have consistently identified classical risk factors for the development of DFU, including neuropathy, peripheral artery disease (PAD), foot deformities, history of ulceration or amputation, nephropathy, poor glycemic control, prolonged diabetes duration, and advanced age [44,45]. Analyses of DFU outcomes remain heterogeneous due to differences in study populations, methodologies, and outcome definitions [45,46,47]. The literature consistently demonstrates that classical predictors (PAD, poor glycemic control, renal dysfunction, neuropathy, ulcer severity, and infection) are strongly associated with adverse DFU outcomes, regardless of study design. In contrast, demographic and systemic factors (age, sex, smoking, obesity, comorbidities) are more frequently highlighted in meta-analyses and large-scale reviews [46,47,48].

This analysis was undertaken to address the clinical challenge of recognizing patients with the highest risk for an unfavorable DFU outcome and which factors are the most relevant for such an outcome. As death as a systemic outcome is different from localized foot outcomes, the analysis of the two cohorts (all subjects and survivors only, respectively) was expected to discern potential differences between these two types of outcomes. Overall, patients with DFU are recognized to carry a markedly elevated mortality risk, a finding reflected in our cohort, where death occurred in 27% of participants in just over 2 years of observation. Meta-analyses report 5-year mortality rates of ~30% for DFU, rising to 46% after minor and 57% after major amputation, with even higher rates in patients with comorbidities, particularly chronic kidney disease [49]. Another meta-analysis estimated 5-year mortality at nearly 50%, with cardiovascular disease and infection as leading causes of death. Consistent predictors of mortality included older age, peripheral arterial disease, chronic kidney disease, previous amputation, and cardiovascular disease [4]. Specific causes of death were not assessed in this analysis.

The frequency of unfavorable outcomes (53% in the whole cohort and 36% when excluding the deaths) underscores the high morbidity and mortality associated with diabetic foot disease. These rates are similar to previously published data, where DFU recurrence alone reaches ~40% at one year, ~60% at three years, and up to 65% at five years after healing [2,12].

Systemic inflammatory markers in this study demonstrated substantial prognostic value for adverse outcomes. Elevated CRP, WBC, and ESR were consistently associated with unfavorable prognosis in both cohorts. These findings are in line with previous studies reporting that elevated WBC and low albumin increase the risk of amputation [46], while reduced hemoglobin and raised CRP, neutrophil-to-lymphocyte ratio (NLR), and platelet-to-lymphocyte ratio (PLR) are linked to limb loss [16]. CRP has been identified as an independent predictor of amputation [19], and large cohort studies further confirmed that elevated CRP and ESR, together with increased WBC and anemia, independently predict adverse outcomes [50].

In this cohort, radiologically confirmed osteomyelitis emerged as a strong prognostic factor for adverse outcomes in the overall population. However, after excluding patients who had died, its predictive significance somewhat decreased and was less pronounced than that of prior amputation as well as BMI, systemic inflammatory markers, or renal function. There may be several explanations for this. First, the presence of osteomyelitis and elevated inflammatory markers may represent a systemic effect, better reflected with the inclusion of death [49]. Second, the lower prognostic value of osteomyelitis may be due to its heterogeneous clinical presentation and the limited sensitivity of radiography, as bone biopsy with microbiological culture remains the diagnostic gold standard [8,51]. Reliance on radiographic criteria alone may, therefore, have underestimated its prevalence and prognostic impact, making it appear less predictive than systemic or host-related factors.

In the present study, triglycerides were the strongest lipid-related predictors of adverse outcomes, exceeding total, HDL, and LDL cholesterol in prognostic value. This is in line with previously published evidence that triglycerides have stronger prognostic value in diabetic foot disease than traditional cholesterol fractions. Unlike LDL-C and HDL-C, triglycerides directly reflect insulin resistance and metabolic dysregulation; additionally, triglyceride-rich remnant lipoproteins contribute to endothelial dysfunction, oxidative stress, and impaired wound healing [52]. Meta-analyses and cohort studies consistently show that elevated triglycerides predict DFU development and amputation risk regardless of diabetes type [53,54,55,56]. We observed a U-shaped association, where both elevated and low triglyceride levels were linked to adverse outcomes: hypertriglyceridemia driving vascular injury and very low levels reflecting malnutrition, catabolism, or severe illness, as similarly reported in cardiovascular and critically ill populations [57,58]. Taken together, these data reinforce our observation that triglycerides may represent a more clinically relevant lipid marker for adverse DFU outcomes than traditional cholesterol fractions in such models, reflecting a broader interplay between metabolic dysfunction, vascular injury, and impaired wound healing.

A history of lower-limb amputation emerged as one of the most powerful predictors of unfavorable outcomes, confirming its role as a critical prognostic factor in diabetic foot disease. The association remained robust in both samples, with its predictive value increasing further in survivors, where it appeared as the strongest predictor. These observations are consistent with prior reports demonstrating that previous tissue loss substantially increases the likelihood of ulcer recurrence, subsequent amputations, and mortality [15,46,59,60]. This emphasizes the need for intensified follow-up and targeted preventive strategies in this particularly high-risk subgroup of patients with diabetic foot disease.

In this study, an obesity paradox pattern was evident: in the survivor-only analysis, lower BMI was consistently associated with worse prognosis, whereas higher BMI appeared to be protective. Poor nutritional status and systemic catabolism may partly explain why lower BMI is associated with worse outcomes in diabetic foot disease. This paradox, widely described in chronic conditions such as chronic kidney disease, heart failure, and chronic obstructive pulmonary disease (COPD), highlights that underweight individuals often experience poorer outcomes due to malnutrition, sarcopenia, and systemic inflammation [61]. Consistently, a recent meta-analysis confirmed that lower BMI significantly increases the risk of amputation in patients with DFU, suggesting that reduced body mass reflects diminished physiological reserve and resilience [46]. Importantly, vascular cohorts have shown that higher BMI may sometimes be protective, likely reflecting greater nutritional reserve and the capacity to withstand systemic stress [62].

Interestingly, shorter diabetes duration and younger age were unexpectedly associated with unfavorable outcomes, particularly in the full-cohort analysis. Although longer diabetes duration and older age are established risk factors for DFU development [60], previous studies have shown that younger patients in tertiary care often present with more severe ulcers, poorer glycemic control, and higher infection rates, reflecting more aggressive disease progression [5,63,64]. Consistently, our patients with shorter diabetes duration demonstrated a similarly adverse risk profile, suggesting that rapid progression to severe DFU may indicate a particularly aggressive phenotype. As this cohort also consisted of tertiary-care patients, referral bias may partly explain the worse outcomes observed in this subgroup.

CKD is an established prognostic risk factor for DFU. Large cohort studies have demonstrated that even moderate reductions in eGFR (<60 mL/min/1.73 m^2^) nearly double the risk of developing DFU, while advanced CKD (eGFR < 30 mL/min/1.73 m^2^) confers up to a four-fold higher risk compared with preserved renal function [65]. The risk of lower-extremity amputation also rises sharply with CKD progression, particularly in stage 4–5 CKD and among patients receiving dialysis [66,67]. In line with these observations, this work identified reduced eGFR as an independent prognostic factor for adverse outcomes in the overall cohort. Dialysis patients were excluded to minimize prognostic confounding, since their survival is often dominated by competing systemic risks. When deaths were censored, the prognostic impact of reduced eGFR became more evident, although it remained less pronounced than prior amputation, BMI, and systemic inflammatory markers.

An interesting observation in our cohort was a weaker but consistent association between metformin use and more favorable outcomes in survivors. This finding aligns with prior evidence suggesting that metformin may reduce the risk of DFU development and progression [68]. The biological plausibility of these findings may relate to metformin’s pleiotropic effects, including AMPK activation, improved endothelial function, attenuation of oxidative stress, and broad anti-inflammatory properties [69,70]. However, it is important to note the potential for confounding by indication: metformin is less likely to be prescribed in patients with multiple comorbidities, particularly in those with advanced chronic kidney disease (CKD), where its use is contraindicated. Conversely, although insulin therapy has previously been associated with incident DFU [71,72], no such association was observed in this analysis, and no other antihyperglycemic agents demonstrated significant effects in any of the cohorts.

The relatively low importance of HbA1c in our models likely reflects several factors. A single baseline HbA1c at the time of ulcer presentation may not accurately capture longer-term glycemic exposure or recent changes in glucose control. Subsequent intensification of diabetes management (e.g., insulin initiation, inpatient stabilization) can further weaken the association between baseline HbA1c and clinical outcomes. In addition, HbA1c is correlated with multiple metabolic variables, and in models that include stronger and more proximate determinants of outcome—such as CRP, radiographic osteomyelitis, WBC count, triglycerides, eGFR, and BMI—the independent predictive contribution of HbA1c diminishes as its shared variance is absorbed by these more direct drivers.

This work had several limitations. First, the retrospective design introduces the potential for residual confounding despite multivariable adjustments. Second, radiographic diagnosis of osteomyelitis may have underestimated its true prevalence, as bone biopsy remains the diagnostic gold standard. Finally, this study was conducted in a single tertiary-care center and involved a limited sample size, which may restrict the generalizability of the findings to broader diabetic populations and, thus, should be regarded as an exploratory analysis. To mitigate the risk of overfitting, we applied internal validation using repeated cross-validation and evaluated model performance using boosting algorithms, including CatBoost and LightGBM. These findings provide preliminary evidence supporting the relevance of these variables, and the modelling framework developed here will need to be expanded and validated in larger cohorts to enable a more reliable estimation of predictive performance and clinical utility. In summary, the routine assessment of triglycerides, renal function, and inflammatory burden, combined with detailed documentation of prior amputations and body composition, could enhance risk stratification in patients with diabetic foot ulcers. Integrating these systemic markers into clinical practice may facilitate earlier identification of frail, high-risk individuals and enable more targeted interventions to reduce amputation rates, ulcer recurrence, and premature mortality.

## 5. Conclusions

In this high-risk cohort with DFU, a prior amputation, systemic inflammation, renal dysfunction, and dyslipidemia were the strongest prognostic factors for unfavorable outcomes. Triglycerides outperformed conventional lipid fractions, while lower BMI and shorter diabetes duration paradoxically indicated worse outcomes, reflecting frailty and accelerated disease progression. These findings emphasize that systemic and host-related factors, rather than local foot features alone, are central to prognosis and should guide risk stratification and management in diabetic foot disease.

## Figures and Tables

**Table 1:** Baseline characteristics of the subjects.

Variable	Mean ± SD	Min–Max	Q1	Median	Q3
Age (years)	63.62 ± 8.79	41.00–77.00	57.00	65.00	69.25
Diabetes duration (years)	20.19 ± 12.19	1.00–60.00	12.00	18.00	26.00
Previous HbA1c (%)	8.50 ± 1.69	5.60–13.40	7.25	8.30	9.60
BMI (kg/m^2^)	32.55 ± 6.96	15.04–54.05	27.85	31.70	36.69
T. cholesterol (mmol/L)	4.49 ± 1.35	2.03–8.60	3.45	4.35	5.35
HDL cholesterol (mmol/L)	1.11 ± 0.37	0.42–2.40	0.86	1.01	1.28
LDL cholesterol (mmol/L)	2.77 ± 0.93	1.25–5.41	2.03	2.60	3.37
Triglycerides (mmol/L)	2.15 ± 2.02	0.39–17.98	1.16	1.65	2.62
eGFR (mL/min/1.73 m^2^)	74.45 ± 24.27	14.10–127.68	59.40	74.90	93.15
WBC (×10^9^/L)	8.68 ± 3.35	4.20–23.20	6.50	7.80	10.10
CRP (mg/L)	47.69 ± 64.59	2.00–290.60	5.60	18.65	56.58
ESR (mm/h)	47.49 ± 33.40	2.00–116.00	15.50	47.00	80.50

Abbreviations: SD—standard deviation; Min—minimum; Max—maximum; Q1—first quartile (25th percentile); Q3—third quartile (75th percentile); Median—second quartile (50th percentile); HbA1c—glycosylated hemoglobin; BMI—body mass index; T. cholesterol—total cholesterol; HDL—high-density lipoprotein; LDL—low-density lipoprotein; eGFR—estimated glomerular filtration rate; WBC—white blood cell count; CRP—C-reactive protein; ESR—erythrocyte sedimentation rate.

## Data Availability

The datasets presented in this article are not readily available because the data are part of an ongoing study. Requests to access the datasets should be directed to the corresponding author.

## Supplementary Materials

The following supporting information can be downloaded at: https://www.mdpi.com/article/10.3390/diagnostics15233070/s1.

## Abbreviations

The following abbreviations are used in this manuscript:

AMPK	AMP-Activated Protein Kinase
AUC	Area Under the Receiver Operating Characteristic Curve
BMI	Body Mass Index
CatBoost	Categorical Boosting
CI	Confidence Interval
CKD	Chronic Kidney Disease
COPD	Chronic Obstructive Pulmonary Disease
CRP	C-Reactive Protein
CV	Cross-Validation
DFU	Diabetic Foot Ulcer
eGFR	Estimated Glomerular Filtration Rate
ESR	Erythrocyte Sedimentation Rate
ESRD	End-Stage Renal Disease
FN	False Negative
FP	False Positive
GLP-1RA	Glucagon-Like Peptide-1 Receptor Agonist(s)
HbA1c	Glycosylated Hemoglobin
HDL-C	High-Density Lipoprotein Cholesterol
ICD-10-AM	International Classification of Diseases, 10th Revision, Australian Modification
IQR	Interquartile Range
IWGDF	International Working Group on the Diabetic Foot
LDL-C	Low-Density Lipoprotein Cholesterol
Median	Second Quartile (50th percentile)
NLR	Neutrophil-to-Lymphocyte Ratio
Q1	First Quartile (25th percentile)
Q3	Third Quartile (75th percentile)
PAD	Peripheral Artery Disease
PDP	Partial Dependence Plot
PLR	Platelet-to-Lymphocyte Ratio
RF	Random Forest
ROC	Receiver Operating Characteristic
SD	Standard Deviation
SGLT-2i	Sodium–Glucose Cotransporter 2 Inhibitor(s)
SHAP	SHapley Additive exPlanations
TC	Total Cholesterol
T2D	Type 1 Diabetes
T2D	Type 2 Diabetes
TN	True Negative
TP	True Positive
TPE	Tree-Structured Parzen Estimator
WBC	White Blood Cell (count)
WHO	World Health Organization
