# Supplementary material for: Predictors of Unfavorable Outcomes in Diabetic Foot Ulcers
Source: Diagnostics (Basel). 2025 Dec 2;15(23):3070. doi: 10.3390/diagnostics15233070 (PMC12691370; doi:10.3390/diagnostics15233070)
Supplement: Supplementary file 1 [file diagnostics-15-03070-s001.zip › diagnostics-3962827-supplementary.pdf]

Supplementary Material

Calculations for Performance metrics

Accuracy shows the proportion of correct predictions out of all predictions and is defined as

$$\text{Accuracy} = \frac{\text{TP} + \text{TN}}{\text{TP} + \text{TN} + \text{FP} + \text{FN}};$$

Precision, given by

$$\text{Precision} = \frac{\text{TP}}{\text{TP} + \text{FP}},$$

measures the proportion of predicted positive cases that are truly positive;

Recall (or Sensitivity) indicates the ability of the model to correctly identify positive cases and is defined as

$$\text{Recall} = \frac{\text{TP}}{\text{TP} + \text{FN}};$$

Specificity measures how effectively the model recognizes true negative cases and is computed as

$$\text{Specificity} = \frac{\text{TN}}{\text{TN} + \text{FP}};$$

F1-score is the harmonic mean of precision and recall, i.e. the trade-off between them. It is useful when both FP and FN are costly. F1-score is defined as

$$\text{F1-score} = 2\frac{\text{Precision} \cdot \text{Recall}}{\text{Precision} + \text{Recall}};$$

Balanced Accuracy is particularly suitable in healthcare datasets, where positive and negative outcomes may be unevenly distributed. It is given by

$$\text{Balanced Accuracy} = \frac{\text{Sensitivity} + \text{Specificity}}{2};$$

Area Under the Receiver Operating Characteristic Curve (AUC) quantifies the model's ability to discriminate between classes across all thresholds. The AUC of 1 indicates a perfect discrimination, while 0.5 indicates no better than random guessing.

Abbreviations:

TP – True Positive, correctly predicted positive cases
TN – True Negative, correctly predicted negative cases
FP – False Positive, negative cases incorrectly classified as positive
FN – False Negative, positive cases incorrectly classified as negative

AUC – Area Under the ROC Curve; measures discriminatory ability across thresholds
